# Decreased insulin resistance in diabetic patients by influencing Sirtuin1 and Fetuin-A following supplementation with ellagic acid: a randomized controlled trial

**DOI:** 10.1186/s13098-021-00633-8

**Published:** 2021-02-05

**Authors:** Mahnaz Ghadimi, Farshad Foroughi, Sima Hashemipour, Mohammadreza Rashidi Nooshabadi, Mohammad Hossein Ahmadi, Mojtaba Ghadimi Yari, Maria Kavianpour, Hossein Khadem Haghighian

**Affiliations:** 1 Department of Nutrition, School of Health, Qazvin University of Medical Sciences, Qazvin, Iran; 2 Department of Immunology, School of Medicine, Qazvin University of Medical Sciences, Qazvin, Iran; 3 Metabolic Diseases Research Center, Research Institute for Prevention of Non-Communicable Diseases, Qazvin University of Medical Sciences, Qazvin, Iran; 4 Department of Pharmacology, School of Pharmacy, Ahvaz Jundishapur University of Medical Sciences, Ahvaz, Iran; 5 Department of Laboratory Sciences, School of Allied Medical Sciences, Qazvin University of Medical Sciences, Qazvin, Iran; 6 Determinants of Chemistry, Amirkabir University of Technology, Tehran, Iran; 7 Department of Tissue Engineering and Applied Cell Sciences, Faculty of Advanced Technologies in Medicine, Tehran University of Medical Sciences, Tehran, Iran

**Keywords:** Ellagic acid, Glycemic index, Insulin resistance, Diabetes type 2

## Abstract

**Background:**

The beneficial effects of polyphenols have been reported. This study aimed to investigate the effect of oral Ellagic acid (EA) supplement on insulin resistance (IR) and Fetuin-A and serum sirtuin1 (SIRT1) in type 2 diabetics.

**Methods:**

In this double-blind, randomized clinical trial, 44 diabetic patients were selected. Patients were assigned to the intervention group (22 subjects) and placebo (22 subjects) and received a capsule containing 180 mg of EA per day or placebo for eight weeks, respectively. At the beginning and end of the study, anthropometric indices, fasting plasma glucose (FPG), plasma insulin level, IR, Fetuin-A, and SIRT1 were measured. Statistical analysis was performed using SPSS software.

**Results:**

At the beginning and end of the study, there was no significant difference between the two groups regarding anthropometric indices (P > 0.05). At the end of the survey, EA supplementation significantly reduced FPG, insulin, IR, and Fetuin-A and increased SIRT1 levels compared with the placebo group (P < 0.05). However, these changes were not significant in the placebo group (P > 0.05).

**Conclusion:**

EA with antioxidant properties plays an essential role in reducing the macrovascular and microvascular complications of diabetes by reducing inflammation and insulin resistance.

*Trial registration* The protocol of this clinical trial is registered with the Iranian Registry of Clinical Trials (http://www.IRCT.IR, identifier: IRCT20141025019669N13)

## Background

Diabetes mellitus is one of the five leading causes of death in the world, characterized by impaired insulin secretion, insulin activity, or both, and is divided into two categories: type 1 diabetes and type 2 diabetes [1]. Results of studies show that in 2030, 552 million people will have type 2 diabetes. Insulin function in diabetics and its subsequent resistance or sensitivity to insulin are the cases that, despite significant research and success in this field, many aspects of it, especially in post-receptor defects, have not been fully identified for the medical research [2]. According to scientific reports, there are factors and cytokines that play a stimulating or inhibitory role in insulin function. Recently, Sirtuin1 and Fetuin-A have been considered by researchers in diabetic patients [3].

Silent information regulator 1 (SIRT1) proteins, known as sirtuins, were identified as genetic silencing factors. Mammalian sirtuins proteins are homogeneous from Sir2 of yeast and prolong the lifespan in yeast [4]. Mammals have seven types of sirtuins from SirT1 to SirT7. SIRT1 is a NAD-dependent histone deacetylase that senses oxidative stress conditions and increases a protective cellular response. To regulate transcription, it transfers acetyl groups from the amino acids *N*-acetyl lysine in DNA histones. It is also expressed in the brain, heart, liver, pancreas, skeletal muscle, spleen, and adipose tissue. SIRT1 plays a vital role in the pathology, progression, and treatment of several diseases, including neurological disorders such as Alzheimer's disease, cardiovascular disease, metabolic disease, and aging-related disease [5].

SIRT1 inhibits oxidative stress through deacetylation of p53, NF-κB, and Forkhead box class O family member proteins (FoxOs) and induction of manganese Superoxide dismutase (SOD). Deacetylation of p53 and FOXO by SIRT1 reduces transcription activity and subsequently reduces stress-induced apoptosis [6]. Scientific investigations have reported that SIRT1 negatively regulates transcription factors such as NF-κB in the nucleus by lysine destabilization on histones and other non-histone proteins. Also, SIRT1 protects cells from oxidative stress by increasing catalase activity. Studies have shown that SIRT1 activity in retinal tissue in diabetic rats is reduced [7]. In cells with insulin resistance, SIRT1 is significantly reduced, resulting in impaired glucose tolerance [8].

Fetuin-A (α2-Heremans-Schmid glycoprotein) A is a 64-kilo daltonic glycoprotein with multiple functions that are produced in the liver and then secreted in the serum. Fetuin-A is a suitable inhibitor of Pathological vascular calcification; however, it is an important factor in insulin resistance [9]. Fetuin-A is located in the 3q27 gene in the human genome, which is a prone site for diabetes and metabolic syndrome. Numerous studies have shown that Fetuin-A levels in patients with T2D and obesity increased compared to the healthy control group. Reports have also shown that Fetuin-A levels are associated with an increased risk of metabolic syndrome. Fetuin-A inhibits the insulin receptor Tyrosine kinase in skeletal muscle and liver cells and stops insulin signals transmission, thereby causing insulin resistance in these target tissues [10]. Fetuin-A in mice with insulin resistance caused by a high-fat diet led to a low regulation of TLR4-mediated inflammatory signaling in adipose tissue [11]. According to the above contents, increasing SIRT1 levels and decreasing Fetuin-A levels is a new therapeutic approach to treating metabolic disorders such as type 2 diabetes. Polyphenols stimulate the expression of SIRT1, which protects cells from oxidative stress [12]. The reason why polyphenols increase the level of SIRT1 in the body is not well known, but it can be related to their antioxidant effect [13].

Ellagic acid (EA) is a polyphenol found in various fruits, including berries, raspberries, pomegranates, and walnuts [14]. Due to this polyphenol’s antioxidant property and the possibility of its favorable effect on SIRT1 and Fetuin-A levels and reduced insulin resistance, this study aimed to investigate the effect of EA oral supplement on SIRT1, Fetuin-A, and insulin resistance in patients with type 2 diabetes.

## Materials and methods

### Ethics

The current research project's approval was done as a randomized, double-blind, placebo-controlled clinical trial by the ethical committee of Qazvin University of Medical Sciences (IR.QUMS.REC.1398.079) and with final registration and approval on the site of clinical trials in Iran (IRCT20141025019669N13). The study was performed on people with type 2 diabetes at the Endocrinology and Metabolism Clinic of Qazvin University of Medical Sciences. According to the Human Studies Protocol, the participants in the research project signed a consent form.

### Inclusion and exclusion criteria

People with type 2 diabetes (hemoglobin A1c (HbA1c) ≥ 6%; or fasting plasma glucose (FPG) ≥ 126 mg/dl (7 mmol/l); or 2-hp glucose ≥ 200 mg/dl (11.1 mmol/l), satisfaction with participation in clinical trials, no change in diet, physical activity and medication were the inclusion criteria. Having a body mass index in the range of obesity, pregnancy and lactation, insulin injection, diseases that interfere with the treatment of diabetes, use of antioxidant supplements, and dissatisfaction with participation in clinical research intervention are the criteria for not including the study. Also, changes in lifestyle (diet and physical activity) and treatment methods, and any reported side effects of the supplement were exclusion criteria.

### Patients

At the beginning of this intervention study, with the scientific opinion of the expert and clinical consultant of the present investigation, 54 people with type 2 diabetes of both sexes, and aged 24–55, a total of 54 people were introduced to the researcher to explain the research process fully and finally enter the study. After complete explanations and additional introduction, 44 people entered the study with complete satisfaction. After complete explanations and introduction of the dietary supplement used in the project, 44 people entered the study with complete satisfaction.

### Study design

To investigate the effect of 180 mg Ellagic acid supplementation daily for 60 days on insulin resistance and serum SIRT1 and Fetuin levels, this randomized, double-blind, placebo-controlled clinical trial study was designed, approved, and performed. Patients who entered the research project were fully informed and justified about the study and its implementation steps, then the required information such as demographic characteristics, medical history, and current drugs as well as anthropometric data [height, weight, and body mass index (BMI)] was collected by the project researcher using a questionnaire and appropriate tools. Grouping of patients in the intervention (EA) (n = 22) and placebo (n = 22) groups was done randomly (by randomized block allocation) and after equalizing their entry in the group in terms of age, sex and weight. Each person received an ellagic acid capsule (180 mg) or a placebo capsule containing wheat flour once daily with meals. The color, shape, and size of the supplement capsule were similar to that of the placebo capsule. In this study, the patient, researcher, and specialist physician were blind to supplement and placebo. Capsules prepared by someone else outside the study in A and B groups were placed in the same package so that the investigator would be unaware of the capsules’ contents. The effective selective dose for Ellagic acid supplement was taken from an article by Mario Falsaperela et al. The supplement was purchased from a SupplementSpot, and the placebo was made by School of Pharmacy, Tabriz University of Medical Sciences. To control for confounding effects of diet and physical activity, at the beginning and at the end of the study, patients were interviewed by a 3-day dietary recall questionnaire, and subjects with moderate physical activity level were enrolled. Three-day food recalls were used to assess dietary intake, and the Nutritionist IV program (San Bruno, CA) modified for Iranian food composition was used for estimating the dietary intake of participants. Also, to evaluate the physical activity, we used the International Physical Activity Questionnaire (IPAQ) [15]. Data from the IPAQ were converted to metabolic equivalent-minutes/week using existing guidelines [16]. Patients were followed up to control their ellagic acid capsules' consumption and prevented from falling out once every seven days by telephone. At the end of the study, each person should return the bottle containing their supplement for count capsules. Patients who consumed less than 10% of capsules were removed from the study.

### Biochemical measurements

At the beginning and end of the study, ten ccs of venous blood samples were taken, and after the separation of serum, SIRT and Fetuin levels were measured in the plasma of the participants. Blood samples were collected into tubes with and without EDTA. Blood samples without EDTA Centrifuged (Beckman Avanti J-25, USA) at a rate of 3000 rpm for 10 min in order to the separation of serum. Blood samples were kept at − 70 °C (Snijdes, Germany) in Diabetes Research Center and then transferred to the laboratory of Velayat Hospital of Qazvin University of Medical Sciences, and the measurements were carried out. Fasting plasma glucose (FPG) concentration was measured by the enzymatic method using an Abbot ModelAclyon 300, USA auto analyzer with Pars-Azmone kit (Tehran, Iran). Plasma insulin was measured by using a chemiluminescent immunoassay method (LIAISON analyzer (310360) Diasorin S.P.A., Verecelli, Italy). Insulin resistance (HOMA-IR) was calculated according to the following formula: HOMA-IR = (fasting insulin (U/ml) × FPG (mg/dl)/405) [17]. Serum levels of SIRT1 and Fetuin-A in participants’ plasma were measured using a special kit and by ELISA method (Diameter, Italy and Bioassay Technology Laboratory, China).

### Sample size

The level of the Malondialdehyde (MDA) factor was used to calculate sample size before and after the administration of pomegranate extract in the study of Hosseini et al. [18] using the following formula:

$${\text{N}} = \left[ {\left( {{\text{Z}}_{{1 - {\upalpha }/2}} + {\text{ Z}}_{{1 - {\upbeta }}} } \right)^{2} \left( {{\text{SD}}_{1} ^{2} + {\text{SD}}_{2} ^{2} } \right)} \right]/\Delta ^{2}$$

where a (type 1 error) is 0.05, b (type 2 error) is 0.2, S1 and S2 are the variances of MDA and ∆ represent the different means of MDA. Thus, the power for detecting differences between the two groups for various outcomes in the present study was 80%. The sample size was obtained, 13 in each group. Considering 30% probable drop-out, the sample size was considered 22. In this research, 44 diabetic patients were studied.

### Statistical analyses

Statistical analyses were conducted using SPSS version 20. Results were presented as mean ± SD, and the normality of data distribution was assessed by the Kolmogorov–Smirnov test. To comparison of mean biochemical variables before and after intervention in each group was used paired sample t-test statistical method and to compare the variables between two groups was used independent sample t-test method. Differences were considered statistically significant at P < 0.05.

## Result

In this study, 44 people with type 2 diabetes participated, which were divided into two groups of 22 people (intervention and placebo). In this investigation, one patient from each group was excluded from the study for personal reasons. Patient compliance in this study was 95.45%. The duration of this study was eight weeks. No side effects were reported in the study. Patients’ individual characteristics are shown in Table 1. There is no statistically significant difference between the two groups in terms of individual characteristics. The mean ages of participants in the intervention and placebo groups were 47 ± 5.06 and 44.04 ± 6.88 years old, respectively (P > 0.05). Also, there was no significant difference between the two groups in terms of anthropometric factors at the beginning of the study. The mean and standard deviation of weight (67.23 ± 10.24 vs. 68.95 ± 9.02), Height (162.43 ± 10.59 vs. 163.71 ± 9.12), and BMI (25.32 ± 1.07 vs. 25.61 ± 0.86) were in the intervention and placebo groups, respectively. Also, there was no significant difference in the amount of physical activity. The amount of food intake is given in Table 2. There were no statistically significant differences at the beginning and end of the study in terms of energy intake, macronutrients, and micronutrients. The effects of Ellagic acid supplementation on FPG, Insulin, IR, STRT1, and, Fetuin-A are shown in Table 3. There were no statistically significant differences between intervention and placebo at the beginning and end of the study. At the end of the study, EA supplementation reduced FPG, Insulin, IR, and Fetuin-A levels and increased SIRT1 levels (P < 0.05). In the placebo group, the differences in mean levels of SIRT1, Fetuin-A, and IR at the end of the study were not significant.

**Table 1:** The comparison of baseline characteristics of the participants

Variable	Mean ± SD Placebo (n = 21)	Mean ± SD Ellagic acid (n = 21)	P1
Age (years)	44.04 ± 6.88	47 ± 5.06	0.701
Height(cm)	163.71 ± 9.12	162.43 ± 10.59	0.814
Weight (kg)			
Before	68.95 ± 9.02	67.23 ± 10.24	0.621
After	68.49 ± 9.21	66.98 ± 10.31	0.59
P2	0.78	0.619	
Body mass index (K g/m^2^)			
Before	25.61 ± 0.86	25.32 ± 1.07	0.562
After	25.43 ± 0.9	25.22 ± 1.09	0.604
P2	0.719	0.807	
Physical activity			
Before	37.27 ± 2.78	36.15 ± 2.99	0.132
After	37.5 ± 3.31	36.43 ± 2.89	0.108
P2	0.429	0.49	
Metformin dose	1643.17 ± 377.88	1610.4 ± 362.07	0.88

Data are expressed as means ± SD

P1: Comparison of the mean of baseline characteristics between the two groups of Ellagic acid and placebo (Independent samples t-test)

P2: Comparison of mean of baseline characteristics in each group at baseline and end of study (Paired samples t-test)

**Table 2:** The comparison of the dietary intake at the baseline and the end of the study in participants

Variables	Mean ± SD Placebo (n = 21)	Mean ± SD Ellagic acid (n = 21)	P1
Energy (kcal)			
Baseline	2057.71 ± 230.546	2034.41 ± 286.84	0.405
End	2043.49 ± 241.269	2026.38 ± 292.2	0.3
P2	0.58	0.48	
Protein (g)			
Baseline	82.19 ± 16.3	80.81 ± 23.11	0.55
End	81.07 ± 21.03	78.27 ± 18.01	0.41
P2	0.68	0.6	
Carbohydrate (g)			
Baseline	263.61 ± 62.19	260.71 ± 49.13	0.59
End	261.39 ± 51.09	259.14 ± 52.2	0.52
P2	0.63	0.69	
Fat (g)			
Baseline	77.5 ± 16.22	76.3 ± 15.07	0.801
End	75.18 ± 24.18	74.17 ± 15.07	0.705
P2	0.309	0.41	
Saturated fatty acids (g)			
Baseline	24.08 ± 6.31	23.27 ± 6.17	0.77
End	23.1 ± 6.07	22.06 ± 5.17	0.662
P2	0.47	0.6	
Monounsaturated fatty acid (g)			
Baseline	28.09 ± 8.14	26.45 ± 7.3	0.807
End	28.22 ± 7.12	26.65 ± 5.3	0.59
P2	0.63	0.74	
Polyunsaturated fatty acid (g)			
Baseline	25.28 ± 7.64	25.56 ± 7.03	0.82
End	24.15 ± 5.18	24.27 ± 5.17	0.86
P2	0.354	0.29	
Fiber (g)			
Baseline	9.27 ± 1.03	8.95 ± 1	0.48
End	8.65 ± 1.44	8.07 ± 1.2	0.401
P2	0.28	0.25	
Vitamin C (mg)			
Baseline	69.14 ± 20.19	68.47 ± 13.27	0.74
End	67.47 ± 23.01	68.03 ± 14.37	0.52
P2	0.583	0.641	
Vitamin E (IU)			
Baseline	8.24 ± 1.01	7.66 ± 1.24	0.456
End	7.53 ± 1.21	7.87 ± 1.01	0.11
P2	0.353	0.84	
Selenium			
Baseline	121.77 ± 41.02	120 ± 25.07	0.481
End	120.16 ± 44.53	119.44 ± 19.61	0.3
P2	0.347	0.491	

Data are expressed as means ± SD

P1: Comparison of the mean of dietary intake between the two groups of Ellagic acid and placebo (Independent samples t-test)

P2: Comparison of mean of baseline characteristics in each group at baseline and end of study (Paired samples t-test)

**Table 3:** Changes in baseline to endpoint measures for SIRT1, Fetuin-A and HOMA-IR in two groups

Variables	Mean ± SD Placebo(n = 21)	Mean ± SD Intervention(n = 21)	P1
FBS (mg/dl)			
Baseline	163.28 ± 7.90	160.80 ± 8.94	0.811
End	162.52 ± 8.54	123.47 ± 9.73	0.022
P2	0.91	0.011	
Mean changes	− 0.76 ± 0.64	− 37.33 ± 0.79	0.032
Insulin (µU/ml)			
Baseline	16.08 ± 0.77	15.83 ± .88	0.53
End	16.00 ± .84	12.16 ± .96	.031
P2	0.77	0.03	
Mean changes	− 0.08 ± 0.07	− 3.67 ± 0.08	0.039
HOMA-IR			
Baseline	6.49 ± .63	6.30 ± .72	0.45
End	6.43 ± .68	3.72 ± .58	0.038
P2	0.59	0.02	
Mean changes	− 0.06 ± 0.05	− 2.58 ± 0.14	0.042
Fetuin-A (µg/ml)			
Baseline	123.58 ± 24.11	119.8 ± 25.07	0.21
End	121.63 ± 24.01	101.31 ± 19.07	0.031
P2	0.159	0.022	
Mean changes	− 1.95 ± 0.1	− 18.49 ± 6	0.033
SIRT1 (ng/mL)			
Baseline	6.04 ± 0.04	5.96 ± 0.05	0.46
End	6.02 ± 0.03	6.94 ± 0.09	0.033
P2	0.66	0.01	
Mean changes	− 0.02 ± 0.01	+ 0.98 ± 0.04	0.046

Data are expressed as means ± SD

P1: Comparison of the mean of dietary intake between the two groups of Ellagic acid and placebo (Independent samples t-test)

P2: Comparison of mean of baseline characteristics in each group at baseline and end of study (Paired samples t-test)

## Discussion

This study was designed to investigate the effect of EA on insulin resistance, SIRT1, and Fetuin-A in patients with type 2 diabetes. This study showed that the administration of 180 mg of ellagic acid for eight weeks significantly reduced blood sugar, insulin, and insulin resistance. Babaian et al. Observed that daily intake of 240 ml of unsweetened pomegranate juice reduced insulin resistance in patients with type 2 diabetes. In contrast, no significant changes were observed in blood sugar and fat levels. Low doses of EA in pomegranate juice or short study time are the reasons for the lack of significant effect on glycemic indexes [19]. Also, Kanchana et al. studied the anti-diabetic effect of EA in diabetic mice caused by streptozotocin for 35 days. The results of their study showed that rats with diabetes had a significant increase in plasma glucose, HbA_1_c, and hexokinase levels and decreased insulin and peptide C, glycogen (liver and muscle), and glucose 6 phosphatase activity in the liver and kidneys compared with healthy mice. Oral administration of EA for 35 days significantly reduced blood glucose levels, HbA1c, the enzyme hexokinase, glycogen (liver and muscle), and the activity of glucose 6 phosphatases in the liver and the kidneys [20]. Based on the reported results of the scientific studies, it has been suggested that a possible mechanism for lowering blood sugar may be by enhancing insulin secretion from pancreatic beta cells or by transferring more blood glucose to the surrounding tissue [21]. In diabetic animals, EA may increase insulin secretion or glucose uptake [20]. Insulin resistance as a lack of sensitivity to insulin function in skeletal muscle, liver, and adipose tissue. After consuming carbohydrates, insulin stimulates glycogenesis in muscles and the liver and inhibits lipolysis in adipose tissue [22]. When IR occurs, the altered insulin response results in the secretion of free fatty acids from adipose tissue and the storage of triglycerides in the muscles and liver. Oxidative stress and activation of inflammatory pathways and lipolysis caused by macrophages promote insulin resistance and non-alcoholic fatty liver disease (NAFLD). Advances in IR can lead to dyslipidemia, high blood pressure, excessive blockage of blood vessels, and fibrinolysis disorders. IR increases the level of oxidized-LDL and cardiovascular disease incidence, particularly in patients with type 2 diabetes [23].

Also, administering 180 mg of EA for eight weeks significantly increased SIRT1 and reduced Fetuin-A in patients with type 2 diabetes. Reducing excess body fat causes a significant increase in the levels of this enzyme [24]. Studies have shown that lowering SIRT1 in mice impairs glucose tolerance. Mice fed a high-fat diet have lower levels of SIRT1 in the pancreas and liver and may be associated with insulin resistance. Inhibition of SIRT1 disrupts insulin signaling by interfering with the phosphorylation of the insulin receptor and glycogen synthase [25]. Khalili et al. assessed the Lactobacillus casei effects on Glycemic response, Serum Sirtuin1, and Fetuin-A Levels in Patients with Type 2 Diabetes. They reported that the levels of SIRT1 and Fetuin-A were significantly affected by probiotic consumption. In their investigation, the L. casei supplementation significantly increased SIRT1 and decreased Fetuin-A levels at the end of the trial [3]. SIRT plays an essential role in many biological processes, including metabolism, cell proliferation, tumor suppression, genomic stability, and aging prevention. One way to reduce oxidative stress in the body and create balance is to increase SIRT secretion [26]. By increasing the expression of antioxidant and anti-inflammatory enzymes and reducing pro-inflammatory factors such as NF-KB, this enzyme ultimately benefits the body by increasing its antioxidant capacity and reducing the destructive effects of ROS [4]. SIRT1 deacetylates lysine 310 of RelA/p65 subunit of NF-kB, an important subunit for for activation of transcription of proinflammatory genes, triggers inflammatory processes.This NF-kB signaling pathway is the prototypical one involved in inflammation [27, 28]. In this way, it can be said that by affecting the secretion of this enzyme, it can help treat diabetic patients by reducing insulin resistance [7, 29]. Janda et al. showed that serum Fetuin-A levels were associated with blood sugar deposition in the arteries and that Fetuin-A had been shown to affect the AGE/RAGE pathway in the presence of inflammatory molecules [30]. Excessive Fetuin-A expression increases levels and activates NF-κB and other inflammatory cytokines. Fetuin-A levels are also positively correlated with CRP levels. This protein is activated by free fatty acids (FFAs) and triggers pre-inflammatory reactions [31]. In addition, in order to promoting insulin resistance in type 2 diabetic patients, Fetuin-A can also prevent the change of RAGE ligand with (HMGB1), which is responsible the release and absorption of several cytokines, adhesive molecules, and cheomokines. Activation of the RAGE signal cascade pathway has been shown to balance HMGB1 expression in tumor necrosis factor (TNFa) and interleukin 1 (IL-1) [32].

Having parallel groups to eliminate the effect of the drug and controlling confounding factors such as physical activity and diet are the strengths of the study. Also, according to the research of the researchers of the present study, supplementation with pure EA was performed for the first time on the IR index. However, it should be noted that in order to determine the clinical effects and cost-effectiveness of this supplement in type 2 diabetic patients, it is necessary to have more participants and study several doses, because in this plan due to existing limitations such as financial, the number of participants and the unit dose studied, the results of this study are statistically interpreted.

## Conclusion

In conclusion, the results of this study indicated that eight weeks of supplementation with EA, 180 mg/day, reduced the levels of blood sugar, Insulin, IR, and Fetuin-A and increased SIRT1 in patients with type 2 diabetes. These results provide evidence to support the view that polyphenol antioxidant group with reducing the complications of diabetes can play an important role in helping to control the condition of diabetes, including reducing the dose of medications used. Nevertheless, further studies are needed to provide additional evidence.

## Data Availability

The datasets used and/or analyzed during the current study are available from the corresponding author upon reasonable request.

## Abbreviations

BMI: Body Mass Index
DM: Diabetes mellitus
EA: Ellagic acid
FPG: Fasting blood sugar
FoxOs: Forkhead box class O family member proteins
HbA1c: Hemoglobin A1c
IPAQ: International Physical Activity Questionnaire
IR: Insulin resistance
MDA: Malondialdehyde
NAFLD: Non-alcoholic fatty liver disease
ROS: Reactive oxygen species
SIRT1: Sirtuin1
SIRT1: Silent information regulator 1
SOD: Superoxide dismutase
